# Long-Standing Overt Ventriculomegaly in Adults (LOVA): Can You Blame Alcohol?

**DOI:** 10.7759/cureus.59973

**Published:** 2024-05-09

**Authors:** Abhinav Kadam, Parav Tantia, Prajakta Kakde, Sunil Kumar, Sourya Acharya

**Affiliations:** 1 Department of Medicine, Jawaharlal Nehru Medical College, Datta Meghe Institute of Medical Sciences (Deemed to be University), Wardha, IND; 2 Department of Medicine, Jawaharlal Nehru Medical College, Wardha, IND

**Keywords:** normal pressure hydrocephalus, long-standing overt ventriculomegaly in adults (lova), chronic alcoholism, macrocephaly, neuropsychiatric effects of alcoholism

## Abstract

Long-standing overt ventriculomegaly in adults (LOVA) is a kind of chronic hydrocephalus that has been reported to have started in infancy and is characterized by severe ventriculomegaly and macrocephaly. It often manifests clinically in later adulthood. We describe the case of a 34-year-old male patient who had a history of chronic alcoholism and who had been complaining of headaches, disturbed gait, and frequent falls for three months when he arrived in a stupor at the emergency room. Massive ventriculomegaly with Evans' index of 0.40 was found during a head magnetic resonance imaging (MRI). The MRI results were more severe than the clinical manifestations. He was diagnosed with LOVA and treated with conservative hyperosmolar drugs, neuroprotective agents, and intravenous (IV) thiamine. The patient was discharged and consented to follow-up after a hospital stay of seven days.

## Introduction

A chronic hydrocephalus known as long-standing overt ventriculomegaly in adults (LOVA) is characterized by macrocephaly with immense enlargement of the third and lateral ventricles. It is believed to have originated in infancy and presents very mildly in maturity [1]. LOVA often goes undetected and undertreated even at centers of excellence because of its poorly known etiology and various clinical and radiological diagnostic criteria that coincide with other chronic cerebrospinal fluid (CSF) disorders [2].

According to available data, the features of LOVA include urine incontinence, subnormal IQ, macrocephaly, and abnormalities in gait, which are more common in later years of life [3]. LOVA has many of the same signs and symptoms as idiopathic intracranial hypertension and idiopathic normal pressure hydrocephalus (iNPH). An important subgroup of chronic hydrocephalus in adults (CHiA) is idiopathic normal pressure hydrocephalus (iNPH), which is characterized by enlarged cerebral ventricles, urine incontinence, gait apraxia, and cognitive impairment [4].

The variability of ventriculomegaly spans from benign examples to severe illnesses. The four usual presentations are incidental observations, highly symptomatic patients, moderate symptoms including headache and nausea, and late symptomatic cases approaching normal pressure hydrocephalus (NPH) [5]. Additionally, seizures may point to underlying pathophysiology [6,7]. In this instance, we describe a 34-year-old male patient who had been experiencing headaches, abnormalities in his gait, and frequent falls for three months. The patient was subsequently diagnosed with LOVA, the cause of which was disputed to be chronic alcoholism.

## Case presentation

A 34-year-old male patient came to the emergency department in a stuporous state with chief complaints of headaches, gait disturbances, and frequent falls for three months. The patient was a chronic alcoholic for 15 years. The patient used to consume 5-8 drinks of alcohol per day, each containing 30 mL, which has approximately 20 g of alcohol in it, thus comprising a total of 150-200 mL of alcohol every day (daily consumption of alcohol around 100-150 g) for the last one year. The last intake of alcohol was stated in the morning of the same day, approximately three hours back. The patient did not have any comorbidity and did not take any medication daily. The patient was admitted and immediately shifted to a high-dependency unit (HDU) for further management.

On examination, the patient's pulse was 80 beats per minute (bpm), blood pressure was 110/70 mmHg, and oxygen saturation was 98% on room air. There were no signs of pallor, icterus, clubbing, cyanosis, lymphadenopathy, or pedal edema. On systemic examination, the cardiovascular and respiratory system examination was within normal limits. The abdomen examination did not reveal anything significant. Upon the central nervous system examination, the patient was stuporous, responding to deep pain stimuli. Deep tendon reflexes such as plantars were found to be extensors bilaterally. The pupils were bilaterally normal in size and reacting to light.

All biochemical and pathological investigations were sent, which came back as described in Table 1.

**Table 1:** The laboratory investigations of the patient on admission g/dL, gram per deciliter; micron, micrometer; pg, picogram; cumm, cubic millimeter; fL, femtoliter; mg/dL, milligram/deciliter; mEq/L, milliequivalent/liter; IU/L, international units/liter; U/L, units/liter; MCHC, mean corpuscular hemoglobin concentration; MCV, mean corpuscular volume; MCH, mean corpuscular hemoglobin; RBC, red blood cell; WBC, white blood cell; RDW, red cell distribution width; APTT, activated partial thromboplastin time; INR, international normalized ratio; SGOT, serum glutamic oxaloacetic transaminase; SGPT, serum glutamic pyruvic transaminase; HIV, human immunodeficiency virus

Laboratory parameter	Results	Normal values
Hemoglobin	13.6 g/dL	11-14 g/dL
MCHC	33.4 g/dL	32-36 g/dL
MCV	91 micron	79-92 micron
MCH	30.4 pg	27-31 pg
Total RBC count	4.91 × 10^6 ^cells/cumm	2.50-5.50 × 10^6 ^cells/cumm
Total WBC count	5300 cells/cumm	4000-11000 cells/cumm
Total platelet count	1.78 × 10^6 ^cells/cumm	1.50-4.50 × 10^6 ^cells/cumm
Hematocrit	44.6%	40%-54%
Monocyte	4%	2%-8%
Granulocyte	55%	40%-60%
RDW	13.6 fL	12.2-16.1 fL
Eosinophils	1%	1%-4%
Basophil	0%	<1%
APTT	30.6 seconds	29.5 seconds
Prothrombin time	12.7 seconds	11.3 seconds
INR	1.07	1.00
Urea	9 mg/dL	6.24 mg/dL
Creatinine	0.4 mg/dL	0.59-1.04 mg/dL
Sodium	139 mEq/L	135-145 mEq/L
Potassium	3.3 mEq/L	3.5-5.1 mEq/L
Alkaline phosphate	73 IU/L	75-124 IU/L
SGOT	46 IU/L	8-45 IU/L
SGPT	39 IU/L	7-56 IU/L
Total protein	7.6 g/dL	6.0-8.3 g/dL
Albumin	4.3 g/dL	3.4-5.4 g/dL
Total bilirubin	0.7 mg/dL	0.1-1.0 mg/dL
Conjugated bilirubin	0.1 mg/dL	0.1-0.4 mg/dL
Unconjugated bilirubin	0.6 mg/dL	0.2-0.6 mg/dL
HIV card test	Negative	-
Serum amylase	176 U/L	30-120 U/L
Serum lipase	61 U/L	13-78 U/L

**Figure 1:**
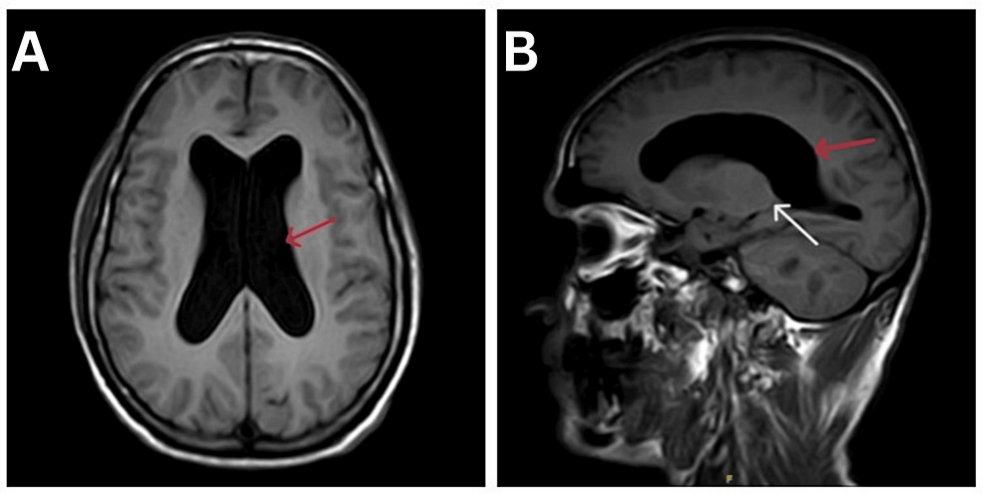
The T1-weighted sequence of the patient's brain MRI (A) Axial section and (B) sagittal section, the red arrows suggesting ventriculomegaly and the white arrow showing aqueductal stenosis MRI: magnetic resonance imaging

The patient was sent for a brain magnetic resonance imaging (MRI), which reported the prominence of sulcal spaces, ventricular system, Sylvian fissure suggestive of ventriculomegaly, and normal pressure hydrocephalus with Evans' index of 0.40 (Figure 1).

**Figure 2:**
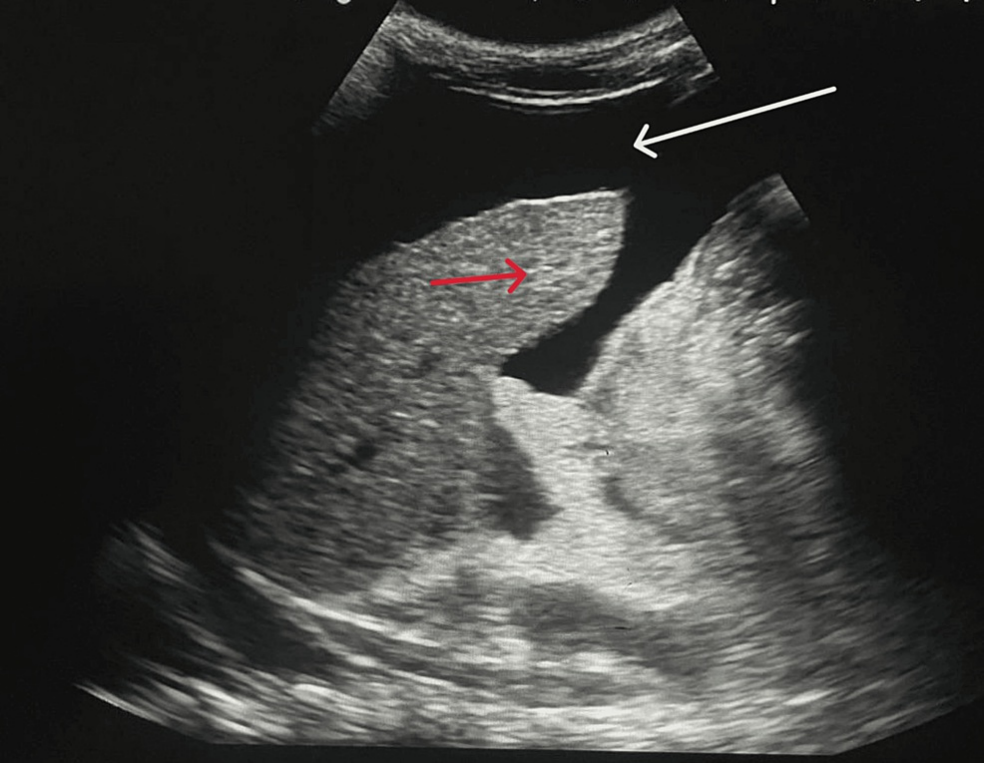
Ultrasound of the abdomen of the patient suggestive of hepatomegaly with raised echotexture of the liver (red arrow) showing grade II fatty liver and moderate ascites (white arrow)

Ultrasonography was done, which was suggestive of hepatomegaly with raised echotexture of the liver showing grade II fatty liver (Figure 2). An electrocardiograph suggested normal sinus rhythm with no ST-T segment abnormalities.

An ophthalmologist opinion was taken for raised intracranial pressure (ICP) features, which suggested no signs of papilledema. A neurosurgery opinion was taken, which indicated no active intervention was required at that time and suggested continuing the same line of management. After establishing a LOVA diagnosis, he was treated with injection citicoline and injection mannitol for neuroprotection. The treatment of chronic alcohol use disorder consisted of thiamine replacement. High doses of intravenous (IV) thiamine, 500-1500 mg, were given thrice daily. Electrolytes and vitamins, in particular magnesium, were also replenished as they were found to be depleted as well in patients with alcohol use disorder. A psychiatrist's opinion was taken to address the problem of addiction for managing withdrawal states in the future, and psychiatric counselling was done as well. The patient was educated adequately on medication adherence, as well as the need for neurological follow-up to monitor his prescription serum levels and seizure activity. The patient's symptoms were relieved, headache and frequent falls were controlled, and the patient did not complain of gait disturbances. He was discharged after a hospital stay of seven days. The patient understood and agreed to follow up as an outpatient. The patient showed up to the outpatient department approximately 15 days after discharge and stated complaint of mild headache intermittently. He was advised for a brain MRI on follow-up but could not do it due to financial restraints.

## Discussion

LOVA, which was first reported by Oi et al. in 2000, is a distinct type of chronic hydrocephalus with probable infant-onset hydrocephalus that presents with symptoms in adulthood. It is characterized by macrocephaly with a head circumference greater than two standard deviations, severe ventriculomegaly, and/or signs of the sella turcica being destroyed or expanding [3]. Several compensatory mechanisms, such as alternate circulation pathways, have been proposed to restore CSF flow before the fusion of the cranial fontanelles, leading to the development of macrocephaly and supratentorial ventricular dilation. The symptoms of hydrocephalus are thought to manifest only after these compensatory mechanisms wear out in adulthood [2].

The heterogeneity of adult ventriculomegaly underscores the varied clinical presentations, ranging from asymptomatic cases to those with significant symptoms [5,8]. Additionally, LOVA's unique trajectory, originating in childhood and manifesting later in life, contributes to its silent progression until clinical manifestations emerge [9-11]. On the other hand, it is well documented in the literature that chronic alcoholism leads to complaints such as headaches, gait disturbances, frequent falls, and other features of normal pressure hydrocephalus [12,13]. Many of these symptoms overlap with those of LOVA.

This variability highlights the need for individualized diagnostic and management strategies tailored to each patient's needs. This is especially pertinent in cases where patients remain asymptomatic and stable despite undiagnosed ventriculomegaly or present in an emergency scenario. In the case presented, the patient with a history of chronic alcoholism experienced a decline in consciousness and multiple neurological symptoms, prompting further evaluation to uncover a potential underlying cause.

The absence of focal neurological deficits, urinary incontinence, or visual changes complicated the assessment of this case. Nevertheless, despite unremarkable physical examination findings and a negative history of any neurological complaint in childhood or adolescence, radiological imaging revealed severe ventriculomegaly. Coupled with a history of alcohol consumption and the patient's age at the time of first presentation, this raised suspicion of an atypical presentation of LOVA as a possible diagnosis. This isolated case is a valuable addition to the existing body of knowledge on LOVA, contributing to our understanding of its diverse clinical manifestations.

This case emphasizes the need for a comprehensive approach to evaluating patients with alcohol use disorder, considering both clinical symptoms and radiological findings. Despite the absence of typical features associated with hydrocephalus, such as focal neurological deficits, the presence of severe ventriculomegaly on imaging, coupled with reported gait disturbances and the patient's age at the time of symptom onset, raises suspicion of an atypical presentation of LOVA.

Further research and clinical studies are warranted to enhance our understanding of LOVA, to confirm the relationship between alcohol consumption and LOVA, and to refine diagnostic criteria and management strategies. Expanding our knowledge base and considering atypical presentations such as the one described here can improve patient outcomes and provide more targeted and effective care for individuals with hydrocephalus.

## Conclusions

The presented case highlights the complexities involved in diagnosing and managing hydrocephalus, particularly in adult patients. While iNPH is a well-known subtype within CHiA, LOVA presents a distinct clinical entity with unique features and challenges. The variability in clinical presentations and the silent progression of LOVA from childhood to adulthood underscore the importance of individualized diagnostic and management strategies tailored to each patient's needs. Hydrocephalus has a plethora of etiologies, all of which need to be managed accordingly. It is imperative to determine probable causes, such as genetic mutations, ageing, alcohol consumption, sleep apnea, hypertension, electrolyte disturbances, and congenital processes, to determine the best possible treatment options. We put forth a case of LOVA for which alcohol as an etiological factor could not be ruled out when consumed for long durations. Further research is required to confirm the cause-effect relationship between the two.
